# Application of serum SELDI proteomic patterns in diagnosis of lung cancer

**DOI:** 10.1186/1471-2407-5-83

**Published:** 2005-07-20

**Authors:** Shuan-ying Yang, Xue-yuan Xiao, Wang-gang Zhang, Li-juan Zhang, Wei Zhang, Bin Zhou, Guoan Chen, Da-cheng He

**Affiliations:** 1 Department of Respiratory Medicine, Second Hospital of Xi'an Jiaotong University, Xi'an, 710004, China; 2 Key Laboratory for Cell Proliferation and Regulation Biology Ministry of Education, Bejing Normal University, Bejing, 100875, China; 3 General Thoracic Surgery, Second Hospital of Xi'an Jiaotong University, Xi'an, 710004, China; 4 Department of Surgery, University of Michigan Medical School, MI, 48109, USA

## Abstract

**Background:**

Currently, no satisfactory biomarkers are available to screen for lung cancer. Surface-Enhanced Laser Desorption/ionization Time-of- Flight Mass Spectrometry ProteinChip system (SELDI-TOF-MS) is one of the currently used techniques to identify biomarkers for cancers. The aim of this study is to explore the application of serum SELDI proteomic patterns to distinguish lung cancer patients from healthy individuals.

**Methods:**

A total of 208 serum samples, including 158 lung cancer patients and 50 healthy individuals, were randomly divided into a training set (including 11 sera from patients with stages I/II lung cancer, 63 from patients with stages III/IV lung cancer and 20 from healthy controls) and a blinded test set (including 43 sera from patients with stages I/II lung cancer, 41 from patients with stages III/IV lung cancer and 30 from healthy controls). All samples were analyzed by SELDI technology. The spectra were generated on weak cation exchange (WCX2) chips, and protein peaks clustering and classification analyses were made using Ciphergen Biomarker Wizard and Biomarker Pattern software, respectively. We additionally determined Cyfra21-1 and NSE in the 208 serum samples included in this study using an electrochemiluminescent immunoassay.

**Results:**

Five protein peaks at 11493, 6429, 8245, 5335 and 2538 Da were automatically chosen as a biomarker pattern in the training set. When the SELDI marker pattern was tested with the blinded test set, it yielded a sensitivity of 86.9%, a specificity of 80.0% and a positive predictive value of 92.4%. The sensitivities provided by Cyfra21-1 and NSE used individually or in combination were significantly lower than that of the SELDI marker pattern (P < 0.005 or 0.05, respectively). Based on the results of the test set, we found that the SELDI marker pattern showed a sensitivity of 91.4% in the detection of non-small cell lung cancers (NSCLC), which was significantly higher than that in the detection of small cell lung cancers (*P *< 0.05); The pattern also had a sensitivity of 79.1% in the detection of lung cancers in stages I/II.

**Conclusion:**

These results suggest that serum SELDI protein profiling can distinguish lung cancer patients, especially NSCLC patients, from normal subjects with relatively high sensitivity and specificity, and the SELDI-TOF-MS is a potential tool for the screening of lung cancer.

## Background

Lung cancer is, at present, the most common malignancy in the world and its overall 5-year survival rate is only 14% [1]. The poor prognosis is due largely to lack of sufficient screening and early diagnostic tools to physicians. Currently in clinic the screening and early diagnosis of lung cancer relies mainly on chest X-ray, low-dose computed tomography, bronchoscopy, sputum cytology, and tumor markers including carcinoembryonic antigen (CEA), cytokeratin-19 fragments (Cyfra21-1), carbohydrate antigen 19-9 (CA19-9), squamous cell carcinoma antigen (SCCAg) and neuron-specific enolase (NSE), etc [2]. All these methods, however, lack adequate sensitivity and/or specificity [3-6]. Thus, it is urgent to search for better methods which provide more valuable information for screening and early diagnosis of lung cancer. Because of the marked heterogeneity of lung cancer [5], a panel of biomarkers for screening and diagnosis would be most appropriate. Surface-Enhanced Laser Desorption /ionization Time-of-Flight Mass Spectrometry (SELDI-TOF-MS), an innovative proteomic technology introduced by Hutchens and Yip [7], has overcome many of the limitations of two-dimensional electrophoresis and Matrix-Assisted Laser Desorption/ionization Time-of-Flight Mass Spectrometry (MALDI-TOF-MS) [8,9]. This is a high through-put technique for analysis of complex biological specimens such as serum. It can detect multiple protein changes simultaneously with high sensitivity and specificity [10,11]. Recently, SELDI has been successfully used to distinguish pancreatic, ovarian and prostate cancer patients from controls [9,12,13], and detect markers of bladder cancer in urine [14].

The aim of the current study was to investigate the application of serum SELDI protein profiling to distinguish lung cancer patients from a healthy population.

## Methods

### Patients

A total of 208 serum samples including 158 pathologically confirmed lung cancer patients and 50 healthy subjects were collected from the Department of Respiratory and Thoracic Surgery of the Second Hospital of Xi'an Jiaotong University. Informed consent was obtained from every subject prior to the study. All patients with lung cancer were found to have no evidence of other disease. The distribution of clinical stages (UICC, 1997) was as follows: 13 cases were at stage I, 41 stage II, 58 stage III, 46 stage IV. Among these patients, 68 patients suffered from squamous cell carcinomas, 53 from adenocarcinomas, 35 from small cell cancers and 2 from bronchioloalveolar carcinomas. The average age of the patients (101 males, 57 females ranging from 28 to 79 years) was 56.8. The healthy controls (31 males, 19 females ranging from 30 to72 years) came from general physical examinations, and had an average age of 54.5. The two groups were matched for age, sex and smoking history. Two milliliters of whole blood were collected during fasting and stored within one hour at 4°C. The blood was later centrifuged for 20 min at 4000 rpm, distributed into 100 μl aliquots, and stored at -80°C until used.

### SELDI protein profiling

Five μL of 10 mM HCl was applied to a weak cation exchange (WCX2) chip and placed at room temperature for 10 min. Chips were rinsed with deionized water in a conical tube and then put into a bioprocessor and washed with binding buffer (100 mM NaAc, pH4) with gentle shaking twice for 5 min. Five μL of each serum and 10 μL of 9 mol/L urea were combined and vortexed on ice. 5 μL of this mixture was added to 60 μL of binding buffer. 50 μL of the serum mixture was applied to each spot and incubated on a shaker for 60 min. Chips were washed again with binding buffer with slight shaking 3 times. 200 μL of 1 mM HEPES pH7.0 was added to each well. Wells were quickly rinsed and then removed and let dry. Once dry, 0.5 μL of sinapinic acid (SPA) was applied to each spot twice. The arrays were allowed to air-dry and then stored in the dark at RT until SELDI analysis.

### Data analysis

Before analysis, the data were randomly divided into two sets as follows: the training set consisted of 11 patients with stages I/II lung cancer, 63 patients with stages III/IV lung cancer and 20 healthy controls. The blinded test (in which the disease status was unrevealed) set consisted of 43 patients with stages I/II lung cancer, 41 patients with stages III/IV lung cancer and 30 healthy controls. The chips were placed in the Protein Biological system II-C mass spectrometer reader (Ciphergen Biosystems, Inc.) and TOF spectra were generated by averaging 128 laser shots with an intensity of 215 and a detector sensitivity of 9. The optimization range was from 3,000 to 50,000 Da, and a maximum of 200,000 Da. External calibration of the instrument was performed using the All-in-one peptide molecular mass standard (Ciphergen Biosystems, Inc.). We achieved a mass accuracy of 0.1% with this system.

### Peak detection

Peak detection using Ciphergen Biomarker Wizard software 3.0.2 identified an average of 72 peaks/spectrum. Of the 72 peaks, 64 common peaks or clusters were generated from the training set. Eighteen of these proteins were found to have statistically differential expression levels between lung cancer and normal control sera (*P *< 10^-4^). Peak detections involved baseline subtraction, mass accuracy calibration, and automatic peak detection. The settings used for our work were as follows: for peak detection the signal-to-noise ratio was 3, minimum peak threshold was 10%; for cluster completion, the cluster mass was 0.5% and the signal-to-noise ratio for the second pass was 1.

### Decision tree classification

Construction of the decision tree classification algorithm was performed by Ciphergen Biomarker Pattern software version 5.0. Classification tree, selected Gini, split the data into two nodes using one rule at a time in the form of peak intensity. The splitting decisions in this case were based on the normalized intensity levels of peaks from SELDI protein expression profile. The process of splitting was continued until terminal nodes were created. After V-fold cross validation 50, the accuracy of each classification tree was then challenged with the blinded test set.

### Detection of serum Cyfra21-1 and NSE

The two markers, Crfra21-1 and NSE, were measured in the 208 sera included in this study using an electrochemiluminescent immunoassay (ECLIA, Elecsys 2010 system, Roche Diagnostics, Switzerland). The cutoff values for Crfra21-1 and NSE, recommended by the manufacturers, were 3.3 ng ml^-1^and 16.3 ng ml^-1^, respectively.

### Statistical analysis

Comparison of relative peak intensity levels between groups was made using the Student's *t *test and in all cases *P *< 10^-4 ^was considered statistically significant. Comparison of rates between groups was conducted using the χ^2 ^test and *P *< 0.05 was regarded as a significant difference.

## Results

### Reproducibility

The reproducibility of each SELDI proteinchip assay was determined by SELDI profiling of 10 aliquots of pooled normal serum. The average coefficient of variance (CV) based on 10 pooled normal human sera for intensities of 22 randomly chosen peaks was less than 20%. Little variation with day-to-day sampling and instrumentation or chip variations was found.

### Serum SELDI profiles of lung cancers versus healthy controls

**Figure 1:**
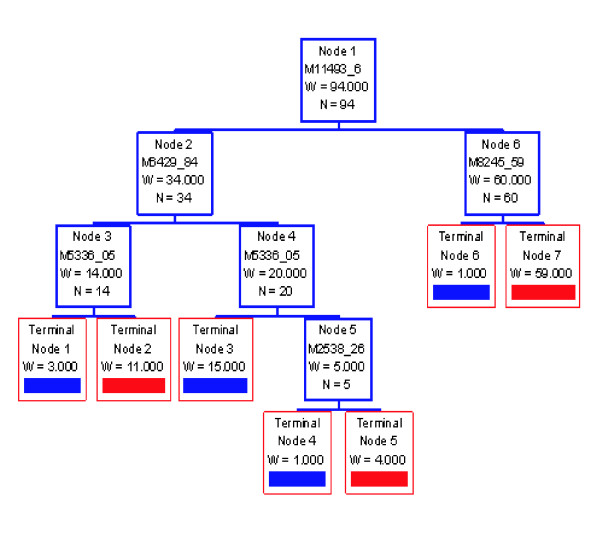
**Classification of lung cancer *vs*. normal samples by the decision tree algorithm. **The left branch node after the first layer is the cases of peak intensity under 2.018, the right one is over or equal to 2.018. The cutoff points for 8245, 6429 and 2538 Da were 1.574, 49.64 and 13.01, respectively. The cutoff points of mass 5335 Da were 0.288 (left) and 2.163 (right). N represents the number of samples. M represents the molecular weight.

**Figure 2:**
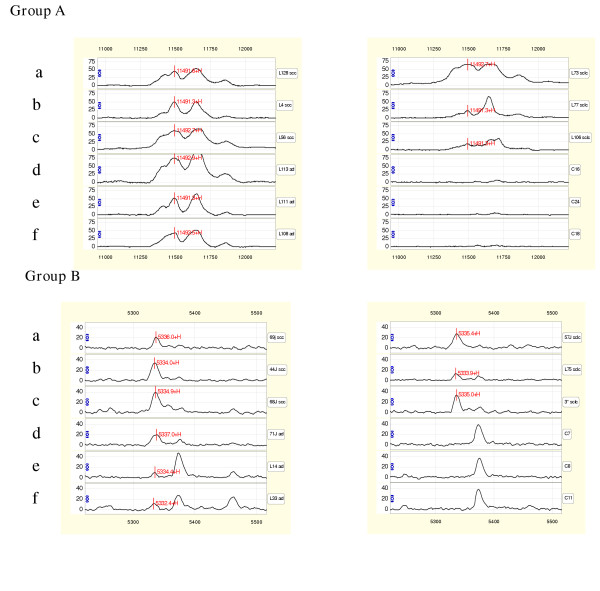
**Differential expressions of the SELDI peaks at 11493 (group A) and 5335Da (group B) in the comparisons of lung cancer and healthy control sera. **a-c: squamous cell carcinomas; d-f: adenocarcinomas; g-i: small cell carcinomas; j-l, healthy controls. X-axis was molecular weight of peak; Y-axis was intensity of peak.

Using Ciphergen Biomarker pattern software to analyze the data derived from Ciphergen Biomarker wizard software, approximately 64 peaks per spectrum identified in the training set were determined with masses ranging from 3–30 kDa. We found that no single peak could adequately discriminate lung cancer sera from normal sera. Using all 64 peaks, a decision tree classification algorithm was built and five protein peaks at 11493, 8245, 5335, 6429 and 2538 Da were automatically selected as splitters. The 11,493 Da peak was used as the root node in the classification tree to divide the 94 samples into two groups (Fig. 1): the left node (node 2) included cases with peak intensity < 2.018. The right node (node 6) contained the remaining with peak intensity = 2.018. The cases in each branch node were then reclassified at the next layer following the same process with 6429, 5335, 2538 and 8245 Da as splitters. This splitting process stops if terminal nodes for further splitting have no gain. Finally, all 94 cases in the training set were classified in the 7 terminal nodes, and a classification tree was obtained (Fig. 1). The tree correctly classified 95.9% of the lung cancer sera in the training set (Table 1). The validity of this classification tree algorithm was then challenged with the test set and a total of 80.0% of controls and 86.9% of lung cancer samples were correctly identified (Table 1). Based on the results of the test set we calculated the sensitivity of the SELDI marker pattern in the detection of lung cancers with different stages and pathological types (Table 2). The peaks at 11493 and 5335 Da are shown in Fig. 2. Aside from the 11493 Da peak, any of the other 5 peaks could have been used as the first node in the classification trees in the same way as 11493 Da, but their performance scores were inferior to the 11493Da peak.

### Discriminatory power of serum Cyfra21-1 and NSE

Table 3 provides the results of sensitivities and specificities of Cyfra21-1 and NSE used individually and combined. We compared the diagnostic capacities of the SELDI marker pattern with Cyfra21-1 and NSE individually and combined (Table 3).

## Discussion

Currently, there are no satisfactory screening and early diagnostic strategies for lung cancer. SELDI is a high through-put technique used to generate protein expression profiles which, in combination with bioinformatics tools to extract information for biomarker discovery, has been essential in identifying novel protein biomarkers. Indeed, application of this technology has shown great potential for the early detection of ovarian and prostate cancers [10,12].

Proteomic studies of lung cancer are still scarce [15]. Recently, Xiao, et al [16] reported that a proteomic panel consisting of three protein peaks yielded a sensitivity of 93.3% and specificity of 96.7% in distinguishing lung cancer patients from healthy controls. This study was, however, based on only 45 tumor samples. In the present study, we examined 158 serum samples from lung cancer patients and 50 from healthy individuals using the SELDI technique with the WCX2 proteinchip. The classification tree was constructed to distinguish lung cancer cases from healthy individuals using 5 protein peaks at 11493, 6429, 8245, 5335 and 2538 Da as a marker pattern. When the model was tested with the blinded test set, it yielded a sensitivity of 86.9%, specificity of 80.0%, and positive predictive value of 92.4% (73/79). For comparison, Cyfra21-1 and NSE were measured using ECLIA in our study. Although there is no statistical differences between the specificities of Cyfra21-1, NSE and the SELDI marker pattern, the sensitivity achieved by Cyfra21-1, NSE individually or in combination were significantly lower than that of the SELDI pattern. These results indicate that the SELDI pattern is distinctly superior to Cyfra21-1 and NSE individually or combined in distinguishing lung cancer patients from healthy individuals.

Based on the results of the blinded test set, we found that the sensitivity of the SELDI marker pattern for NSCLCs was significantly higher than for SCLCs, indicating that the pattern may be more effective in discriminating NSCLC patients from healthy controls than SCLC patients. Similarly, the pattern also had a sensitivity of 79.1% in the detection of lung cancers with stages I/II, suggesting that the pattern might be better for early detection of lung cancer than any other single or panel of biomarkers currently used in clinic [17,18].

To develop a broad biomarker panel for screening a diverse, high-risk population, both NSCLC and SCLC patients were chosen for our study. Due to the relatively fewer healthy control samples and the subgroup of patients with SCLC, our results require more samples to broaden and improve its diagnostic value. Furthermore, the five proteins included in the SELDI marker pattern will be identified by MALDI-MS-MS.

## Conclusion

We have found that serum SELDI protein profiling can distinguish lung cancer patients, especially NSCLC patients, from healthy controls with relatively high sensitivity and specificity. The SELDI-TOF-MS is a potential tool for the screening of lung cancer.

## Competing interests

The author(s) declare that they have no competing interests.

## Authors' contributions

SY was responsible for the conception and design of this study, providing samples and clinical data, drafting and revising the article. XX contributed to the design of this study, performed statistical analysis and interpretation of the data. LZ provided technical support and some experiments. WZ and BZ provided in part study materials and medical aspects of the work. GC has been involved in discussion and revising this article. DH contributed to the conception of this study. All authors read and approved the final manuscript.

## Pre-publication history

The pre-publication history for this paper can be accessed here:



## Figures and Tables

**Table 1:** Classification tree analysis of the lung cancer training and blinded test sets.

Sets	Groups	Percentage correct	Percentage misclassified
Training set	Normal (n = 20)	90.0 % (18/20)	10.0% (2/20)
	Cancer (74)	95.9% (71/74)	4.1% (3/74)
Blinded test set	Normal (30)	80.0% (24/30)	20.0% (6/30)
	Cancer (84)	86.9% (73/84)	13.1% (11/84)

**Table 2:** The sensitivity of the SELDI marker pattern in lung cancer patients with different stages and pathological types.

Variables	n	Correct cases	Error cases	Sensitivity (%)	*P *value
Clinical stages					
I/II	43	34	9	79.1	
III/IV	41	39	2	95.1	<0.05
Pathological types					
NSCLC	70	64	6	91.4	
SCLC	14	9	5	64.3	<0.05

NSCLC: non-small cell lung cancer; SCLC: small cell lung cancer.

**Table 3:** The sensitivities and specificities of the SELDI marker pattern, Cyfra21-1 and NSE.

Items	Pattern	Cyfra21-1	NSE	Combination
Blinded test set				
Sensitivity (%)	86.9 (73/84)	44.0(37/84)^a^	36.9(31/84)^a^	63.1(53/84)^a^
Specificity (%)	80.0 (24/30)	70.0(21/30)	73.3(22/30)	53.3(16/30) ^b^
Whole set				
Sensitivity (%)		46.2 (73/158)	35.4 (56/158)	62.0 (98/158)
Specificity (%)		72.0 (36/50)	74.0 (37/50)	56.0 (28/50)

Pattern: SELDI marker pattern; Combination: combined use of Cyfra21-1 and NSE. ^a ^P < 0.005 compared with the SELDI marker pattern; ^b ^P < 0.05 compared with the SELDI marker pattern.
